# The Chicago Fire

**Published:** 1871-10

**Authors:** 


### THE CHICAGO FIRE.

We do not refer here to the great fire to give any general intelligence in reference to it, but to speak of it as it affects our professional brethren in Chicago; and we can not tell this better than by giving a letter just received from Dr. C. L. Smith, of Springfield, Ills., in which he says: "I have just returned from a visit to the scene of the terrible calamity in Chicago. A great majority of the Dentists there are burned out, their offices at least, and many have lost everything.

Drs. Dean, Cushing and Crouse, are burned out entirely. Dr. Cushing lost everything, not even setting eyes on his office. Dr. Dean saved a few instruments, and a trunk with a little clothing. Dr. Crouse saved considerable of his furniture and instruments, though he lost heavily. The latter two have rented rooms together, and I suppose are able to start afresh. Of all those of whom I learned, Dr. Cushing is in the worst fix; having lost everything, he must start *de novo*." Dr. Cushing is well and favorably known to the whole profession, and we have simply to suggest to each and every one of our readers that they do in these cases as they would have others do to them in similar situation. The profession should see to it that their brothers are put upon their feet again for business.

Let something be done by every one and it will be a light matter, and yet do great good. We trust that attention will be given to this matter in every city and town in the country. Contributions may be sent to either of the parties by mail. T.

